# Perceived healthcare stigma among patients in opioid substitution treatment: a qualitative study

**DOI:** 10.1186/s13011-021-00417-3

**Published:** 2021-10-26

**Authors:** Lars Garpenhag, Disa Dahlman

**Affiliations:** 1 Center for Primary Health Care Research, Department of Clinical Sciences, Malmö, Lund University/Region Skåne, Sweden, Box, 503 22 Malmö, Sweden; 2 Faculty of Medicine, Department of Clinical Sciences Lund, Psychiatry, Lund University, Lund, Sweden

**Keywords:** Drug use, Opioid substitution treatment, Healthcare disparities, Stigma, Sweden

## Abstract

**Background:**

People with substance use disorders (SUD) including patients in opioid substitution treatment (OST) are subject to stigma, and have generally poor health and barriers towards seeking healthcare. Experience of stigma might negatively affect healthcare seeking, but this topic is sparsely investigated. The aim of this study was to explore OST patients’ past and present experiences of substance use stigma in healthcare settings, in order to provide insight into the challenges that people with opioid use disorder may face when using health services, and the strategies they use to cope with them.

**Methods:**

Six focus groups with 23 OST patients were moderated by OST staff, and conducted with a questioning route focusing on health literacy. Experiences associated with stigma and its consequences that were spontaneously brought up by participants were assessed in a secondary analysis using a thematic approach.

**Results:**

Experiences of stigma from a wide range of healthcare settings were reported. Medical records and patients’ oral information regarding substance use, OST medication or hepatitis C infection were identified as circumstances bringing unwanted attention to the SUD. Participants reported various forms of poor treatment, believed to reflect views of people with SUD as morally culpable, intimidating, curious, untrustworthy and less valuable than other patients, sometimes with tangible effects on the quality of healthcare. Stigma in healthcare settings affected healthcare seeking behaviors, and could result in patients concealing their OST status or substance use history.

**Conclusion:**

This study highlights several aspects of perceived healthcare stigma that can shed light on difficulties that OST patients might experience when navigating the healthcare system. The results implicate a need to investigate attitudes towards OST patients, and the aptitude to deal with patients with SUD, among healthcare professionals, as well as a need for interventions addressing knowledge deficits and issues tied to values and patient reception among healthcare staff.

**Supplementary Information:**

The online version contains supplementary material available at 10.1186/s13011-021-00417-3.

## Background

People with substance use disorders (SUD) in general, and opioid use disorder (OUD) in particular, are at risk of a range of adverse health effects. These negative effects include increased drug related and non-drug related mortality [1, 2]; physical morbidity such as hepatitis C, chronic obstructive pulmonary disease and self-reported somatic symptoms [3, 4]; and under-utilization of healthcare [3, 5, 6]. In addition, people with SUD also face stigma and discrimination. Studies have traced a number of adverse outcomes to stigma, ranging from decreased wellbeing, self-efficacy and self-esteem, to avoided or postponed healthcare seeking and lower compliance [7–11]. The use of narcotics and other psychoactive substances have been increasingly medicalized, i.e., understood as a medical problem. However, in large parts of the world, this has not made moralistic values surrounding SUD obsolete [12–14]. A typical example of this in-complete medicalization process is how the use of non-medically-prescribed narcotics often falls under a double jurisdiction, and is treated both as a health problem and as a criminal offence.

Contacts with health services are one instance when people with SUD risk encountering stigmatizing attitudes and discrimination [7, 8, 15–21]. It has been established that the presence of stigma in healthcare contexts is destructive, “negatively affecting people seeking health services at a time when they are at their most vulnerable” [22, page 1]. While stigma is a part of life for persons with a wide range of mental disorders, studies show that the stigma associated with SUD is particularly severe [14, 23]. This might be because of widespread beliefs about culpability, attributing greater moral blame to persons with SUD than to persons suffering from many other health problems [14, 24, 25]. It has also been suggested that substance use stigma has a greater impact on the lives of people with SUD than other grounds for discrimination such as race, sex and poverty [20].

Importantly, stigma related to drug use does not necessarily cease to affect individuals as they go into remission or enter treatment [26]. On the contrary, opioid substitution treatment (OST) has been found to be surrounded by substantial stigmatizing attitudes, potentially affecting both patients and the professionals who treat them [13, 20, 27–34].

Despite the critical need for more knowledge on how people in OST experience and react to stigma in healthcare settings, research on this subject is sparse. The great majority of previous studies on the topic have been conducted in the US, while the situation in other countries has been explored to a far lesser extent. Sweden forms a setting that is markedly different from the US in important respects, combining universal, tax-financed healthcare services that are strongly subsidized for residents [35] with a notoriously restrictive drug policy, which criminalizes use [36]. Drug-induced fatalities in Sweden are the highest in Europe (81 deaths/million, with opioids involved in a majority of the cases) [37].

In a mixed-methods study of the health literacy in OST patients in Malmö, Sweden, perceived poor reception and treatment were reported as factors that negatively affected communication with healthcare professionals, and thus their access to both healthcare and health information [38]. The fact that stigma and discrimination proved important to the participants’ experiences of health information and services gives further merit to the case that such themes deserve to be further explored. Through a secondary analysis of data, this qualitative study explores OST patients’ past and present experiences of substance use stigma and poor reception in greater detail, concentrating on healthcare settings outside of specialist SUD care. The aim is to provide insight into the stigma that people with opioid use disorder (OUD) may experience – or anticipate – as well as the strategies they use to cope with it. The analysis addresses a range of questions pertaining to the beliefs, feelings, actions and reactions of patients, but also to the concrete contexts and situations in which patients experience stigma.

## Methods

### Theoretical framework

In this paper, we study stigma related to SUD and OST treatment on the individual level, as experienced by OST patients, focusing on their perceptions of stigma in healthcare settings, and their attempts to manage it. Our understanding of stigma is based on Weiss, Ramakrishna & Somma’s definition of health-related stigma as:

typically a social process, experienced or anticipated, characterized by exclusion, rejection, blame, or devaluation that results from experience or reasonable anticipation of an adverse social judgment about a person or group [ … ] based on an enduring feature of identity conferred to by a health problem or health-related condition [39, page 280].

In short, our analysis concerns OST patients’ experiences of negative social repercussions that comes with being identified as a person with SUD, or as an OST patient, in interactions with the healthcare system. This includes subjective experiences of poor treatment and discrimination, as well as anticipations regarding the risk of becoming subjected to it, and a more general awareness of how people with SUD are subjected to negative stereotypes and judgments – what is commonly referred to as perceived stigma, or stigma awareness [40].

As highlighted by Link and Phelan, stigmatizing processes are dependent on power. For an attitude or action to be stigmatizing, it has to be situated in a power relationship in which the active part is actually able to discriminate the individual or group subjected to it. Thus, while negative attitudes harbored by healthcare professionals towards their patients could fruitfully be considered as stigmatizing, patients’ attitudes towards professionals cannot be viewed in the same way [41], at least not unless there are other power relations at play in the interaction between them.

The significance of medical legitimacy to stigma is an important aspect of the concept that has been the subject of different interpretations in the literature. While Weiss, Ramakrishna and Somma argue that medically warranted judgements should not be considered stigmatizing [39], this is clearly not how stigma has been understood in previous research about SUD and OST, in which, e.g. repressive practices integral to OST-treatment have been described as stigmatizing or discriminatory without regard to their underlying medical rationale [13, 28]. Similar to Matthews, Dyer and Snoek in their study of substance use stigma [11], we disregard the question of justification in determining what falls within the purview of stigma.

### Study design and setting

We used qualitative methods to conduct a secondary analysis of focus group data collected in a previous study about health literacy in OST patients in Malmö, Sweden. The focus group interviews took place at an OST clinic in Malmö. The clinic is a subsection to Addiction Center Malmö, a public healthcare service provided by Region Skåne (the county council of Skåne). The choice of clinic was motivated by practical considerations concerning the availability of space and staff resources needed to conduct the interviews.

With c. 350,000 residents, Malmö is one of Sweden’s largest cities, and also a part of the transnational metropolitan Öresund region, with a population of four million. At the time, the OST clinic was one of six in the city, serving a total of approximately 540 patients. Compared to the rest of Sweden, Region Skåne has high OST availability.

In Sweden, OST is provided at specialized psychiatric treatment facilities that can be run by either public or private service providers. OST clinics in Region Skåne are obliged to provide not only pharmacological treatment, but also psychiatric, psychological and psychosocial treatment, infectious diseases testing and simpler forms of somatic healthcare. At the time of the interviews, the OST clinic also provided on-site primary healthcare for their patients.

### Data collection

Data were collected in the fall of 2019, through focus group interviews with patients at the OST clinic. Recruitment was carried out by clinic staff, who individually approached potential study participants. There was also written information about the study displayed at the OST clinic. Participants were provided a grocery store gift card valid for approximately USD 20 as a thank you for their contribution. Ethics approval for the study was obtained from the Swedish Ethics Board (file nr 2019–02680).

Potential participants who were unable to provide informed consent (due to poor language skills, severe substance influence or psychiatric problems), who suffered from psychiatric problems that would act as a barrier to participation or who were deemed to pose a security threat, were excluded from participation. All participants took part in both the oral and written study information, and provided their informed consent. Due to there being a regulated minimum age for enrolment in OST, all participants were aged 20 or over.

A total of six interviews were held, in Swedish, with 3–6 participants in each group (*n* = 23; 13 males). To make for a comfortable discussion climate [42], groups of different gender make ups were held (one all-female, two all-male, and the rest mixed). A counsellor at the clinic arranged the focus groups and moderated the interviews, while one of the authors (L.G.) handled the recording, kept notes and occasionally asked for more details or clarifications. The interviews lasted between 30 and 60 min, were recorded digitally, and transcribed *in verbatim*. The interviews were based on a questioning route centered on different aspects of health literacy, e.g. participants’ experiences of navigating the healthcare system, how they perceive the information from official healthcare sources, and how they evaluate health information. Questions were created using the concept of health literacy presented by Sørensen et al. [43], and were evaluated by staff at the Malmö Needle Exchange as a part of the preparations for the study. None of the questions pertained directly to stigma or discrimination. Rather, these topics were spontaneously brought up by participants when answering more general questions about health information and healthcare encounters. Please see Additional file 1 for the questioning route.

### Data analysis

A secondary analysis was carried out using a thematic approach [44]. In a first step, the concept of stigma presented above was applied to identify which parts of data were relevant to the present study. All relevant data was coded manually in Microsoft Office Word 2016, and the codes were then developed in a largely inductive reading and re-reading of participants’ statements. Finally, the research questions were used as a thematic framework to sort the codes. Coding and thematic analysis was carried out by one of the authors (L.G.) and reviewed by the other (D.D.). Rather than an elaborated theoretic reinterpretation of the data, the resulting analysis amounts to a “low interference” qualitative description [45, 46], relatively close to the opinions and language of the participants themselves. All quotes have been translated from Swedish to English and edited for clarity by the authors (L.G. and D.D.). To protect their identities, all participants have been given fictitious names reflecting their genders.

## Results

Throughout the interviews, participants spoke extensively about judging attitudes, poor treatment and discriminatory actions that they had endured in interactions with healthcare services. This included experiences they had made both while using illicit opioids and other substances, and while being enrolled in OST.

### Healthcare settings and involved professionals

Participants had experienced poor reception in a diverse range of healthcare settings. These included both inpatient and outpatient health services (e.g. primary healthcare centers and emergency rooms [ERs]); and somatic as well as psychiatric healthcare.

I came to the ER, I suffered from sepsis and all kinds of stuff. Busted head … But then we arrive at what you just mentioned: <in a snarky tone> “Do you take any meds? Oh, you’re in OST, well then you have to go see your OST physician.” That one I’ve heard a hundred times.

*Diana*

When I had my psychosis [ … ] and wasn’t positive on anything [literal meaning “did not have traces of any drug in urine screening test” but can also be interpreted as “had not taken any drugs”], a woman in the waiting room asked – I still remember how she looked – <in an aggressive tone> “What are you on?” I was like “What the hell are you saying?” Well, she meant, what’s your addiction.

*Johanna*

Some accounts clearly concerned events in a healthcare setting, but were unspecific on the particular branch. One participant explicitly argued that for him, it was inconsequential where in the healthcare system you are.

I’ve actually never been in contact with [psychiatric services], but it doesn’t matter where it is, the primary healthcare center, the hospital, the ER … Not even if it is the fucking X-ray clinic you know.

*Petter*

To some participants, the differences between individual healthcare professionals and their characteristics seemed to be more important than the setting. Participants in one of the focus groups proposed professionals’ cultural backgrounds as key to understanding the hostile attitudes they had encountered. Making a difference between personnel with Swedish and non-Swedish backgrounds, they associated prejudice with what they believed to be harsh moral views on illicit drug use in some cultures. However, most participants were strikingly non-specific in their references to professionals, mentioning only very general information like type of workplace, profession or gender.

The people are the problem. Yes, it can be both positive and negative, some can be so like “ah, it’s the addiction, it’s the addiction!” While others … like now I am lucky to have a great primary healthcare physician who knows about my background and everything.

*Sofia*

*Ergi*: They have their own … how you say, culture, in their heads. About something that we here in Sweden, like drug addiction, classify as an illness. And then you have a physician from [participant’s parents’ home country]. He just thinks: “You bastard, you should have a kick in your behind and nothing else. Be taken by the ear.” You know, he doesn’t … it is not an illness to him. It is …

*Karim:* Self-inflicted.

*Ergi*: Yes, it is self-inflicted and you’re an idiot and an inferior human being.

### Circumstances bringing unwanted attention to the SUD

Several participants touched on the question about what had given them away as people with SUD or OST participants in interactions with healthcare, pointing toward a number of ways in which their current or past relationship to opioids and other substances had come to the attention of healthcare professionals. The most obvious one was through having a history of substance use documented in one’s patient records. Since primary and secondary healthcare services in the region keep separate patient record systems, it remained possible to be enrolled in OST (or to be subject to other kinds of SUD care) without primary healthcare providers having any knowledge about it. A few participants recalled – sometimes regretfully – having disclosed such information anyway.

I mean, I have not taken any of my main … you know any drug that has made me unstable so to speak, in almost ten years. I self-medicated [ … ] before I got my diagnosis on paper but I mean, it is still the first thing they notice. It is the first thing they notice.

*Petra*

Being on OST medications was believed to influence interactions with healthcare professionals negatively as well. As with historic substance use, the matter of a patient’s OST enrolment was not always known to all healthcare services beforehand, but could become an issue because they felt obliged to disclose it.

I sometimes avoid contacting [healthcare services] since, well, it’s enough with all the times you’ve been at the doctor’s, and they ask you – you are always supposed to answer this one – “Are you on any medication?” And when you tell them what kind of medication you’re on, you immediately see a big fucking hole being blown right through the head of this person, and from then on, he or she doesn’t hear anything else.

*Katrina*

A few participants had perceived a significant difference in (non-SUD) healthcare professionals’ reactions depending on which OST drug they were on. While methadone was well-known and therefore to be considered as a sure give-away, other drugs were not as widely familiar, and thus not instantly recognizable as parts of an OUD treatment regime.

*Petter*: Sometimes they ask you about which medications you take, if you’re on any medications. “Yes, methadone”. <In a surprised tone> “Oh, really!”

*Camilla*: Hm, yes. They know that one. But many don’t know what Suboxone is.

*Axel*: No, exactly.

*Petter*: That’s what I figured.

*Camilla*: Suboxone, yeah no.

*Axel*: It is better to mention … Subutex maybe, but Suboxone, that’s past the line [of common knowledge].

A final giveaway was brought up by one participant, who believed that he had been poorly received in the past because of suffering from hepatitis C, a condition often perceived as associated with illicit drug use, and injection drug use in particular.

When I had the hepatitis, and had to declare it in the health questionnaire [at appointments], I noticed that I was treated differently. I have a very hard time dealing with that. I have a very hard time with that [ … ], and I felt that some doctors were [thinking] like “you’re a junkie”.

*Milan*

### Manifestations of stigma

Participants described experiences of mistreatment and discrimination of several different types. Impolite, uncompassionate or patronizing attitudes from healthcare professionals was a common denominator for many of the complaints. While poor treatment was not always unambiguously associated with stereotypical thinking or prejudice about drug use, it seemed common to make such connections. A few participants vividly described a change in reception taking place whenever their SUD became known. Others mentioned meeting condemning attitudes clearly related to moral beliefs about SUD.

You have yourself to blame, no sympathy at all. Plus that it’s self-inflicted. You can go home, you will just hurt yourself again anyway.

*Lena*

[When healthcare professionals learn about your SUD] it all changes. They change, from looking at you with warmth, as if you were a fellow human being, straight to “addict”. And immediately you get the “Yeah, no, but you’re exaggerating [your problems]”.

*Petter*

Several participants brought up that healthcare professionals might perceive people with SUD as intimidating. They had been in situations in which they felt that they had been treated like threats to the safety of healthcare professionals, either physically or as potential spreaders of infectious diseases. In one of the groups, participants light-heartedly mentioned a battery of protective gear (“double gloves”, “an apron”, “a splash guard”) being brought out whenever a patient’s SUD came to attention. Others addressed the subject in a more harsh tone. One woman, who had felt poorly treated by healthcare staff, believed that her SUD had not only made them suspect her of carrying infectious diseases, but also to question her mental state.

It is just like if [healthcare staff members think] "Oh, junkie, dirty!” Or “Infections!”, or maybe, “How is her head?”

*Diana*

Meanwhile, professionals’ attitudes did not have to be perceived as straight-out negative for their actions to be perceived as offensive. For instance, one participant complained over meeting unnecessary curiosity after telling a primary healthcare nurse that he was on OST medication. What bothered him was what he found to be an inappropriate and intrusive interest into a matter that he felt was not only private, but also irrelevant to his current treatment.

*Patrik*: Well I was only supposed to get a toe X-ray. Yes, well then they had written … like you know, “he is on methadone”. I was like, “What does this have to do with my toe!?” [ … ] You know, this was the worst, I actually felt violated, or you know, accused.

*Milan*: Yeah, you use drugs. Yeah yeah.

*Patrik*: Yeah, they are supposed to check on my toe, and it has nothing to do with … And then I had to answer a lot of questions about that. Why I take [methadone] and …

*Milan*: Yeah yeah, I bet she was shocked because you don’t fit her preconception [of a person with SUD].

*Patrik*: But it was none of her business!

A number of participants indicated that they had felt dissatisfaction with the quality of healthcare that they had been provided, or that they had been singled out as less valuable than other patients. Some complained that they were not taken seriously, or that their needs were not a priority, because of their SUD.

And you know, anemia, it has nothing to do with my addiction, if you want to put it that way. They told me, for over two years, to eat broccoli. That’s how it was. “What medicines do you take?” And I answered, Buprenorphine or Subutex were the ones at the time. And then it was just “go home and eat broccoli”.

*Lena*

Healthcare professionals being unable to see past participants’ SUD, and over-attributing both physical and mental ailments to the SUD was a reoccurring source of discontent, and it was believed that this could make other health problems less visible. In connection with this, there were complaints that primary health care physicians had tried to shift responsibility for participants’ health needs to the OST clinic in an unduly way.

The first reactions [when telling her primary healthcare provider about her SUD] were bad. In the sense that the counsellor denied me counselling, because they turned everything into the addiction. So, they argued that if I was in contact [with an OST clinic], they have psychologists there.

*Britta*

Suddenly, everything is about your addiction. “Oh, but it is because of your addiction”. Yes but I’m here now, because of a whole different thing.

*Mona*

Several participants had felt subjected to an unwarranted mistrust, or judged beforehand as “drug addicts” (an expression commonly used by the interviewees) rather than based on their individual merits. One cited reason for such mistrust was that, according to the participants, some healthcare professionals believe that patients with SUD are manipulative and looking to get their hands on prescription drugs, another was that they are considered particularly querulous. Some thought that they had been ‘tested’ by healthcare staff who wanted to see if they were simulating medical conditions, or that unjust terms had been imposed as conditions for them to receive treatment.

Well, you know, they don’t believe you. They assume that you lie about most things.

*Kent*

Then, to hear, you know … they take for granted that you will ask them for narcotics. “You will not get narcotics from me!” No, but I haven’t even asked, I have blocked myself from that, you know it is there written in red [in the patient records].

*Lena*

Deficiencies in trust were believed to have several tangible consequences for the quality of healthcare. A common perception was that it makes it more difficult for patients with SUD than for people in general to obtain prescriptions for analgesics or anxiolytics, and that they have to make do with smaller doses than other patients. Another suggested consequence was that it hurts the communication with healthcare professionals.

The worst, according to me, is that when you’re a junkie, or addicted, you can’t get medication … narcotic medications. [ … ] I think that if you’re in pain, and there are medications that can … painkillers, then you should get them, no matter what.

*Karim*

There is some kind of fear. “Oh, you’re an addict and an OST patient, we don’t dare give you … ” Maybe [they] don’t want to take that responsibility. A lack of knowledge maybe [ … ]. I don’t know what the deal is. But that’s how it is most of the time. “No no”. You have to make do with a little less. Put up with the pain.

*Diana*

### Reactions to stigma

The experience of being poorly received or mistreated had impacted participants in several ways. Negative emotional reactions were common. Participants had taken offence, felt singled out and condemned, or even felt dehumanized. For some, negative experiences affected their attitudes to healthcare services, as well as the level of trust they vested in them. For instance, experiences of feeling rejected by healthcare professionals had led at least one participant to anticipate that she would not be able to get help when it was really needed.

It’s so damned outrageous, humiliating and offensive. You aren’t seen as a human being, but as a fucking object. And people have gotten the idea that they can treat you any way they want to. I believe that if you are to work in healthcare, then you should have some empathy and be able to sympathize with your fellow men, and to understand your patient. Because he is a human being!

*Katrina*

Yeah, the fear [is] that you will not get any help because you are judged beforehand.

*Lena*

In one of the groups, participants discussed the broader issue of how discriminatory treatment risked to negatively affect OST patients’ chances to recover and reintegrate into society. Being mistreated, it was suggested, not only decreases the mental wellbeing of patients but also erodes motivation, and makes the efforts associated with being in OST essentially fruitless. The perception that you were still viewed the same way as before, despite being” clean” (i.e., following OST protocol and only using drugs in compliance with the prescription), was frustrating.

We’re supposed to leave our drug abuse behind us and when we do it, and are supposed to reintegrate into society, then we’re spit upon. Because then, suddenly, you don’t have an addiction any more, but you still have a fucking stamp on your forehead, which you can’t escape. And it doesn’t matter how long you’ve been clean or how far along you’ve come in your recovery, or for how long you’ve been able to work. Because you have that fucking stamp. Always.

*Katrina*

This ambiguous status of OST patients was also reflected in a lack of uniformity in how participants referred to themselves and their peers. While some used terms that highlighted the fact that when in OST, they were no longer illicitly using (e.g., by using terms such as “ex-addict”, “former addict”, “sober drug addict”), others failed to make such a distinction. Attitudes to discriminatory behavior from healthcare staff, however, were more uniform. Almost all participants, who had anything to say on the matter, were deeply critical of discrimination they had experienced themselves, and showed sympathy when listening to co-participants’ stories as well. Although some agreed with certain negative notions about people who use drugs in general, they found it wrong to treat individuals differently because of perceptions about the group as a whole – in other words rejecting what they found to be a stereotyping of people with SUD.

There are many [people with SUD] who’ve fooled [healthcare professionals] as well. But you can’t just assume that everybody is lying, you know. [ … ] Until the opposite is proven, you should be treated the same way as any other human being.

*Ergi*

Still, all were not as unambiguously critical regarding discriminatory behavior against people with SUD. One participant took a more understanding stance, arguing that you have to respect that others might be afraid of you, and that, in the end, you have to take responsibility for your mistakes.

You know, drug addicts are frightening. It is difficult for all the people in society to accept us when we are addicts. They have kids as well, and those kids will grow up. They don’t want their kids to suffer from … Like we have. So, they are afraid. I accept them, I respect them even if they treat me differently.

*Samir*

### Coping with stigma

Participants touched upon several ways in which they had adapted to the risk of facing discriminatory treatment. Secrecy was one common strategy. Rather than being open about their SUD or OST enrolment, several participants had instead tried to hide it in contacts with healthcare services. Others cautioned their peers from being too open about such matters.

It is nothing that I would ever consider mentioning, no.

*Camilla*

No, but that’s how it is, according to my experience, you absolutely should not mention, you know, your history. You should never tell a doctor that you [have a history of drug use].

*Axel*

Another way to deal with anticipated healthcare stigma and discrimination was through avoiding seeking regular medical care – either through staying away from certain services or by abstaining totally.

Yeah, you avoid the primary healthcare center if you can. I’m sorry but you do.

*Patrik*

Now I suffer from panic anxiety on and off, but you know, I don’t seek help for it because I know that I will be treated like a shitty junkie who just wants to get drugs in a legal way.

*Mona*

The avoidance of needed services, however, did not necessarily mean that participants lacked access to healthcare altogether. A number of participants testified that they felt safer in contacts with SUD-specific services than in other healthcare settings. Visits to the OST clinic, the Addiction center or the needle exchange clinic came across as opportunities to get help with health problems in general, turning these establishments into a kind of substitute to other services. Even when such contacts ended with a referral, SUD-specific services seemed to serve as gateways into the healthcare system, offering an environment where at least some participants found that they could air health problems safely and openly, without fear of negative repercussions because of their SUD.

I have had a good response here [at the OST clinic]. If I hadn’t been here it would’ve been more difficult. Just because you can’t really mention that you’re a drug addict [elsewhere].

*Petter*

A less drastic answer to negative experiences was to simply change healthcare provider, in the hope to find a service where one would get a better reception. This was also presented as a strategy to deal with situations when physicians refused to prescribe strong painkillers or anxiolytic medications, something that several participants found to be discriminatory and based on a biased view of persons with SUD.

Well most times [when in need of healthcare] I call the primary healthcare center where I’ve been listed. Hm, and start there. Sometimes when I have been unhappy with that center, since they didn’t treat me like an ordinary person, I changed to another one.

*Adam*

It is because [of difficulties to get medications prescribed] that people go to private doctors and stuff like that, because they can’t get help from [public services]. Ex-addicts go to private doctors and clinics because there they aren’t seen as … There they can start fresh. A clean slate.

*Mona*

Meanwhile, strategies to avoid or counteract stigma did not necessarily appear to be desirable paths of action in the eyes of all participants. The perceived need to circumvent health services that should have been the natural place to take one’s business did not seem unproblematic to everyone, while the concealment of current or past substance use, was recognized as associated with health risks by at least some participants.

I’m thinking that if [healthcare professionals] are to take care of me and administer … if you’re really in pain, they need to know. “He takes 110 milligrams of methadone every day, we can’t give him a shot of morphine and start cutting him.”

*Milan*

## Discussion

While stigma among Swedish OST patients has been examined previously [31–33], to our knowledge, this study is one of the first to qualitatively assess perceived stigma in healthcare settings in greater depth. People in OST constitute an aging population that experience high levels of morbidity and mortality associated with conditions that might have been treatable if discovered in a timely manner, e.g. cancers and cardiovascular diseases [1, 2]. By creating greater knowledge about how patients experience stigma and discrimination, in which situations, and how they adapt, the study paves a way for interventions to increase the quality of healthcare for patients with OUD, and to remove barriers to healthcare.

Participants’ negative experiences originated from settings throughout the healthcare system. The lack of precision regarding the setting in many narratives may suggest that the exact place was of lesser importance in the minds of the participants, i.e. that they experience the problem with stigma as endemic. Likewise, while a few participants presented elaborate ideas about culture as an important explanation to negative attitudes to patients with SUD, the sheer volume of stories that failed to mention healthcare professionals’ cultural backgrounds suggest that a majority of participants did not think of it primarily as a culturally conditioned problem, but a general one. Even though patients’ experiences of specialized SUD care services lie beyond the scope of the current analysis, it is worth noting that participants mentioned poor experiences from such settings as well. However, as reported previously based on the same data [38], and in accordance with observations made in the literature regarding other sub-groups of persons with SUD [21], such services were considered by many to be safe spaces in the healthcare system.

According to participants, their SUD had been exposed mainly through written or verbal information (in contrast to visible stigmata), either previously recorded or disclosed by them during medical examinations. Somewhat surprisingly, no participant mentioned being recognized as a person with SUD by healthcare professionals because of stigmata in a more traditional sense of the term, i.e. through physical marks or by appearance. This result deserves to be viewed with a certain caution, since the data on the topic might have been shaped by the question guide, which was focused on health information and comprehension.

Despite the fact that OST is a well-established, evidence based medical treatment, feelings of being discriminated against had not disappeared upon participants’ entry into the OST program. On the contrary, several participants shared the experience that information about their enrolment in OST could bring about negative reactions as well. This finding harmonizes with those in the international literature on stigma and OST. While it has been proposed that OST patients suffer from a “double stigma” [32], based on our data it is difficult to differentiate between what might be a result of condition stigma tied to their SUD, and what could rather be classified as so-called intervention stigma, tied specifically to participation in the OST program [34]. What is clear, however, is that being on OST medications can be experienced as a burden and thus as something to conceal in contacts with other health services.

The ways in which participants had felt poorly treated varied. Quite a few bad experiences involved notions casting persons with SUD as morally culpable, dangerous, or untrustworthy ‘Others’ and less valuable than other patients. Participants’ reactions made it obvious that such experiences can function as a substantial barrier to healthcare seeking, and perhaps also to social reintegration, as suggested in one group interview.

Several participants found it discriminatory to be denied prescriptions for medications they believed that they needed. While such limitations can be seen as a part of the social control that is necessarily associated with OST, and even be construed as a form of discrimination [30], professionals’ decisions to refuse prescriptions might well be medically sound and taken on individual merits to protect the health of patients. Still, the fact that such denials risk to be perceived by patients as a result of prejudice towards people with SUD highlights an area in which there is room for enhancement of the communication between physicians and patients, for instance through an increased professional sensitivity for how such decisions can be interpreted by patients with SUD.

Another problem that participants described was healthcare professionals focusing too much on their SUD, leading to other health problems being obscured or misinterpreted as somehow related to substance use. Referred to as diagnostic overshadowing, this kind of reductionist gaze on patients and their health is in fact a well-known phenomenon associated with mental health and SUD problems. Diagnostic overshadowing may result in under-diagnosis as well as mistreatment of health issues unrelated to the diagnosis that is shaping the professional’s view of the patient [8, 47]. A related problem that was brought up was a tendency in other services to refer OST patients to the OST clinic for a broad set of health problems. Although it is difficult to judge from participants’ stories if such referrals have actually violated the official division of labor within the healthcare system or not, it is worth noting that similar testimony exists already from the early on in the history of OST in Sweden, and has been interpreted as a part of a more general unwillingness to associate with OST patients [48].

Several participants had abstained from seeking healthcare in the past to avoid stigma. Some had simply changed providers as a response to feeling poorly treated, basically using the freedom to choose provider that is considered an important feature in Swedish healthcare policy. Others had let the anticipation of stigma shape their care seeking patterns in more comprehensive ways, e.g. through avoiding seeking primary healthcare, and instead primarily using SUD-specific healthcare facilities as a proxy for or gateway to the rest of the healthcare system. Aside from the inequity associated with having to resort to such strategies, this could obviously also contribute to delays in presenting oneself for care, and subsequently to diagnosis and treatment.

Another way to circumvent stigma was to conceal one’s SUD or OST enrolment. While such secrecy could seem adequate in the short term, participants themselves correctly observed that it could lead to harm, e.g. because it makes it impossible for physicians to take medications’ compatibility with OST drugs into account [21, 27]. In addition, previous research concerning patients in SUD treatment has found secrecy about one’s SUD to be associated with “a number of indicators of poor functioning”, such as lower quality of life and poorer mental health [26]. Thus, as Link and Phelan note often is the case with attempts to counteract the effects of stigma [41], the same strategies that allowed participants to temporarily avoid uncomfortable interactions with healthcare services, simultaneously created new risks.

The results from this qualitative study have implications for further research. By creating greater knowledge about the situations in which stigma and discrimination are experienced and how patients adapt to them, the study paves a way for interventions to increase the quality of the reception that patients with OUD meet. OST patients’ self-reports of negative experiences of healthcare interactions are alarming, but have to be juxtaposed with other kinds of evidence to give a more thorough understanding of the problem. Not least, more knowledge is needed about the people on the other side of the healthcare encounter. A more rigorous exploration of professionals’ attitudes to SUD, but also their self-evaluated aptitude to work with patients with SUD and their views on stigma as a phenomenon, could further enhance our understanding of the stigma surrounding SUD in healthcare contexts, and be vital to the design of interventions, for instance in the form of educational measures geared towards professionals.

While the data in this study did allow some limited insights into how participants related to substance use stigma, a more focused examination of healthcare related self-stigma – that is, the degree to which OST patients accept and integrate negative stereotypical views about SUD in their own self-image – could prove a fruitful avenue for future research. As has been noted in previous research, reactions to stigma depend on factors such as to what degree a person identifies as belonging to the stigmatized group, or internalize prejudicial notions about it [49]. One could hypothesize that the fragmented identity associated with OST as a treatment modality – a reflection of an ambiguity that is embedded in the organization and clinical discourse of treatment [29, 31] – should greatly influence how patients relate to negative attitudes to SUD, and be reflected in a diversity of reactions.

This study has limitations. The data were not originally created to enable in-depth analysis of stigma, which raises questions about meaning saturation. Also, the secondary nature of the analysis makes it hard and sometimes impossible to differentiate between experiences that participants have made as active substance users, and those made while in OST, and associated primarily with that treatment modality. A data collection process tailored specifically for this study could have amended this, and also made it possible to cover certain aspects of how stigma affects patients more thoroughly, e.g. mechanisms of self-stigma, and also made it possible to explore how stigma related to SUD interacts with other health conditions and social relationships such as class, gender and ethnicity [9, 39, 50]. However, while explicit questions on topics directly related to stigma might have yielded richer data, and provided more detailed insights into participants’ experiences, we are confident that the great volume of statements relevant to the topic found in the data provide a solid basis for the analysis. In addition, it can be considered a strength that participants’ accounts represent what they spontaneously found important to share about their encounters with health services, even without being explicitly questioned about stigma. It is also notable that the patients who met the exclusion criteria in this study (inability to provide informed consent due to poor language skills, severe substance influence or psychiatric problems) might be even more stigmatized than the study participants.

## Conclusions

This study was one of the first to qualitatively assess perceived healthcare stigma among OST patients in greater depth. It identified that OST patients had experienced poor treatment and negative attitudes in a wide range of healthcare settings, due to their status as OST patients or people with SUD. Such experiences affected them emotionally, but were also believed to have manifest negative effects on the quality of healthcare. Anticipated stigma can also affect OST patients’ healthcare seeking behaviours and make them conceal their OST status or substance use history. The study highlights several aspects of perceived healthcare stigma among OST patients that can explain the difficulties this patient group experience when navigating the healthcare system. The results provide a basis for future research regarding substance use/OST stigma from patients’ and healthcare professionals’ perspectives, and stress the need for interventions to make healthcare services more acceptable to OST patients and apt at catering for their specific needs, in order to decrease the high morbidity and mortality in this patient group.

## Supplementary Information

**Additional file 1.** Questioning route for focus group interviews about health literacy [translated from Swedish].

## Data Availability

The datasets used and analyzed during the current study are available from the corresponding author on reasonable request.

## Abbreviations

ER: Emergency room
OST: Opioid substitution treatment
OUD: Opioid use disorder
SUD: Substance use disorder
USD: US dollars
