# Supplementary material for: Perceived healthcare stigma among patients in opioid substitution treatment: a qualitative study
Source: Subst Abuse Treat Prev Policy. 2021 Oct 26;16:81. doi: 10.1186/s13011-021-00417-3 (PMC8549326; doi:10.1186/s13011-021-00417-3)
Supplement: Supplementary file 1 — Additional file 1. Questioning route for focus group interviews about health literacy [translated from Swedish]. [file 13011_2021_417_MOESM1_ESM.doc]

Appendix: Questioning route for focus group interviews about health literacy [translated from Swedish]

1. To begin with, we would like to know your first names, and when was the last time you were in contact with the healthcare system concerning a physical problem. We just want the date, nothing else.
2. When you want to find information concerning physical health matters, for instance if you feel ill, where do you look?
3. From your experience, how does it matter where or from whom you obtain such information?
   *Suggested probes*: In what way? What makes different sources more or less suitable or trustworthy?
4. Thinking about the public healthcare system specifically, how do you go about it when you want to turn to them for advice about the body or physical health problems?
5. When you have been in contact with the public healthcare system, have you usually found the information provided to be intelligible?

*Suggested probes*: when needed, probe for added specificity concerning context (kind of caregiver, situation), the type of information, participants’ opinions about it and what could be done better.

1. Try to remember a specific situation in which you have received information from a doctor, nurse or other healthcare staff, and you had trouble comprehending it. How did you handle it?

*Suggested probes*: What did you think or feel, when you choose to act that way?

1. Now we have spoken for a long while about all kinds of aspects of health information, with lots of valuable contributions to the discussion. Is there something on the subject that we have missed?
2. The interview/discussion is closed. The moderator corrects any misunderstandings that have come up that could lead to harm (e.g. harmful advice about where to find health information). Participants receive thanks, a gift for participating, and a flyer with information on trusted sources to turn to in case they have questions about physical health matters.
